# Choosing One’s Words: Conversational Indirectness and Humor Style in Two Distinct Cultural Groups

**DOI:** 10.3390/bs15030252

**Published:** 2025-02-23

**Authors:** Tanisha Y. Berrios, Dun-Ya Hu, Jyotsna Vaid

**Affiliations:** Department of Psychological and Brain Sciences, Texas A&M University, College Station, TX 77843-4235, USA; tberrios@tamu.edu (T.Y.B.); dunyahu@tamu.edu (D.-Y.H.)

**Keywords:** conversational indirectness, humor styles, self-defeating humor, aggressive humor, cultural differences, Korean, English

## Abstract

We investigated cultural differences in the relationship between conversational indirectness and styles of humor use. Our study compared responses of English first language (L1) users (*n* = 56) and Korean first language users studying in the US (*n* = 32) on the conversational indirectness scale) and the humor styles questionnaire. We found no overall group differences in conversational indirectness. Instead, higher indirectness for interpretation than for production was noted, but only in the English L1 group. This group also showed a positive correlation between interpretation and production scores; no such association was found in the Korean sample. On the humor style measure, scores for affiliative and self-enhancing humor were significantly higher in the English L1 group compared to the Korean group; the English L1 group also showed a positive correlation between these two dimensions, and between self-enhancing and self-defeating humor. Both groups showed low identification with self-defeating and aggressive humor styles. There was a significant positive correlation in the Korean group between these two styles. Finally, an association between conversational indirectness and humor style was noted in each group: in both groups, a significant positive correlation was found between indirectness in production and aggressive humor. Additionally, for the English L1 group a significant positive correlation was found between self-defeating humor and indirectness in production and interpretation. These findings demonstrate cultural differences in humor uses and an intriguing relationship between the tendency to produce linguistic meanings indirectly and uses of humor considered to be less positive.

## 1. Choosing One’s Words: Conversational Indirectness and Humor Style in Two Distinct Cultural Groups

How we interact with one another in everyday life is shaped by a plethora of psychological, social, and cultural factors. Two ways in which individuals differ in their interpersonal communication is in whether intended meanings are expressed or interpreted directly or indirectly, and in whether a serious or a playful style is used. There is by now an extensive empirical and theoretical literature on both of these topics, which points to gender, ethnicity, and language/cultural identity as sources of individual differences in conversational style (e.g., Tannen, 1981) and in humor use in conversation (e.g., Vaid, 2006). 

However, research on individual differences in conversational indirectness has largely been conducted separately from research on the uses of humor in everyday interactions. Might these two dimensions of conversational style be inter-related? After all, whereas some types of humor (mimicry, parody) may be considered direct, other types of humor rely on indirect communication. Indeed, both indirectness as a communication style and the use of certain kinds of humor in conversation rely on a difference between what is said (the surface, literal meaning) and what is meant (the intended meaning, which requires an understanding of the social or cultural context, the relationship between the interlocutors, etc.). In addition, people may be motivated to be indirect in their communication or to be nonserious for similar reasons, e.g., to defuse conflict, to preserve face, etc. Our study, therefore, sought to investigate empirically the potential relationship between conversational indirectness and humor style. Given that previous research on these constructs has also pointed to the importance of culture as a source of individual differences (see Jiang et al., 2019, for a review), we examined conversational indirectness and humor style in two distinct linguistic/cultural groups: Americans (L1 English users) and South Koreans (L1 Korean users studying in the US). 

Before turning to our study, we provide a brief overview of previous relevant research on conversational indirectness and humor style. 

## 2. Conversational Indirectness

Conversational indirectness refers to a form of interpersonal communication in which a person is inclined to express their beliefs or attitudes indirectly rather than directly. Certain settings (work vs. home) may call for directness in communication (Sanchez-Burks et al., 2003). Other research has shown that properties of a language (e.g., the impersonal *se* construction in Spanish), can contribute to speakers of the language being perceived as less direct in their characterization of events, particularly in ways of signaling and interpreting agency (see Cunningham et al., 2010). 

Indirectness as a communication style has also been discussed in relation to the construct of individualism/collectivism (Ting-Toomey, 1988; Triandis, 1989), and the related construct of high- and low-context cultures (Hall, 1976). Individuals from cultures considered high-context or collectivist are thought to prefer an indirect mode of communication, in order to keep harmony in their group or out of a concern for others’ feelings. Rather than a weakness, a preference for not stating messages clearly in these cultures may be construed as a marker of maturity and social competence (Miyahara, 1996). 

In a series of ethnographic studies, Tannen (1981, 1984) observed individual differences in the tendency to speak indirectly or to interpret others’ remarks as indirect. She noted that when presented with written scenarios to interpret, American participants chose a more direct interpretation than Greeks and Greek-Americans. Tannen also noted that there are many paralinguistic cues and non-verbal signals that contextualize the directness of a remark. Her work highlighted the potential misunderstanding that can arise in interactions between individuals with different preferences for conversational directness, as when overt and implied messages are confused (Tannen, 1981). Tannen’s seminal work has spawned a number of cross-cultural investigations of direct vs. indirect communication styles (see Merken et al., 2014, for a review).

In an attempt to quantify differences between individuals in their preferences for a direct or indirect mode of communicating, Holtgraves (1997) developed a conversational indirectness scale (CIS). The CIS was developed through the validation of items that addressed individuals’ tendency towards indirectness in production and interpretation. Indirect production refers to an individual’s tendency to speak indirectly and convey indirect meaning in their remarks. Indirect interpretation refers to an individual’s tendency to believe, look for, and find secondary meanings in others’ remarks (Holtgraves, 1997). Although these are conceptually distinct processes, it is reasonable to expect that people who prefer to convey meanings indirectly may also tend to search for indirect meanings in their conversations with others. 

The communication modes of production and interpretation allow researchers using the CIS to investigate these two distinct, but related ways that individuals may vary in indirectness tendencies. Holtgraves validated the CIS by examining participants’ interpretations and varying levels of directness in response to different situations. He then compared the performance of an American undergraduate sample with that of undergraduates in Seoul, South Korea, who were administered a translated version of the scale. Korean participants showed a significantly higher level of indirectness than the American sample in both communication modes. Holtgraves concluded that individuals from Korea favor indirectness in their conversational style compared to those from the United States, presumably because Korean culture is more collectivist and therefore there is a greater tendency towards indirect communication, as a way of preserving group harmony and face. 

Hara and Kim (2004) extended investigation of conversational indirectness to the domain of individual differences in self construal, as a way of exploring differences within cultures. They found that while there were no significant differences in indirectness on the CIS scale between a sample of Asian students and Euro-American students at the University of Hawaii, individuals who were more interdependent in their self-construal showed a positive correlation with indirectness scores (in interpretation and production alike). By contrast, individuals who were independent in self-construal (i.e., more individualistic) showed a negative correlation with indirectness in production. 

European Americans are typically considered more individualistic and tend to have a more independent self-construal and autonomous self-goals, being motivated to focus on their own uniqueness and agency; cultures that are more collectivist prioritize relational self-goals where individuals are motivated to focus on collective activities (Wang, 2016). Autonomous and relatedness self-goals can be experimentally manipulated (Wang & Ross, 2005), as culture and cultural differences are dynamic and change in response to social and physical contexts (Wang, 2016). Additionally, Chen and Wang (2021) argue that not enough attention has been paid to how indirectness varies within East Asian communication, as most studies compare East-Asian communities to Western communities. 

Indirectness norms are also based on what is considered an appropriate or polite level of directness. Indirectness and politeness are distinct, but related constructs (Marti, 2006). Politeness rules are considered to rely on individual face-saving (Brown & Levinson, 1987), especially in individualist cultures where most politeness research originated. In collectivist cultures, individual “face” is based on group membership and is part of a social relationship (Yu, 2011). Because of this face variability between cultures, politeness norms are not universal across cultures, and the relationship between politeness and indirectness varies between cultures and contexts. For example, being direct can be considered polite or impolite depending on the culture and context of the remark (Yu, 2011). Moreover, politeness is not necessarily optional; in some Asian cultures it is prescribed by the grammar of the language (Yu, 2011). 

## 3. Humor and Communicative Style 

The capacity to perceive and generate humor is a ubiquitous aspect of the way humans communicate (Lopez & Vaid, 2017; Vaid & Ramachandran, 2001). Spontaneous humor in conversation can take a variety of forms, from puns and witty repartee to banter and teasing, joke telling or narrating funny anecdotes, and it uses a variety of techniques, from mimicry and exaggeration to sarcasm and irony (Dynel, 2009; Vaid, 2006). Tannen (1984) noted that engaging in humor in a conversation is experienced as a particularly salient contribution to the conversation, compared to other contributions. The sociologist Ervin Goffman (1967) observed that we interact to gain knowledge of others and to present a self-image for ourselves and others. Joke-telling in conversation provides an opportunity for the joker to gather relevant social knowledge about beliefs and attitudes. As Norrick (2003, p. 1342) observes: “Since jokes often trade on personal problems or slips and socially sensitive topics … they allow the joker to demonstrate a certain tolerance and/or insensitivity, while offering hearers a chance to signal their agreement, shock, resentment, or what have you… Joking works to establish and enhance group cohesion, and serves as a control on what sorts of talk and behavior are acceptable to participants in the interaction”. Like norms for indirectness and politeness, norms about what can be joked about, with whom, and how, are likely shaped by culture (Lu, 2023; Vaid, 2006). 

In a study that extended a US-based study of conceptions of an ideal sense of humor (Crawford & Gressley, 1991), Tosun et al. (2018) asked participants from the US, Turkey, and Iran to describe characteristics of an ideal sense of humor as embodied in a known individual. An analysis of elicited narratives showed that creativity (i.e., being witty, clever, playing with language) was the most frequently mentioned characteristic for all groups. Nevertheless, this characteristic was mentioned significantly more by American participants (64%) than by Turkish (48%) or Iranian participants (45%). The characteristic of *caring* (defined as humor used to change a person’s mood when they are upset) was mentioned equally often by American (37%) and Iranian (34%) participants but hardly at all by Turkish participants (2.5%). In addition, hostility/sarcasm (insulting or destructive humor) was mentioned significantly more by American participants (41%) than by Turkish participants (19%) or Iranians (nearly 0%). Thus, characteristics of an ideal sense of humor show interesting cultural differences, with the creative, generative aspect of humor, and the negative aspect of putting others down, being particularly favored by Euro-Americans. In a discussion of other cross-national comparisons, referencing an individualist vs. collectivist framework, Schermer and Kfrerer (2020) refer to studies that show that Americans and Canadians value aggressive forms of humor to a greater degree than do those from Armenian or Lebanese cultural backgrounds. 

Taking a different approach, Vaid et al. (2004) compared individuals’ perceptions of their own frequency of humor use for particular functions and their perceptions of the frequency with which typical members of their cultural group use humor for the same functions. Participants were recruited from four different cultural groups in the US (Euro-American, African-American, second generation Mexican-American and second generation Asian American) and were administered a 19-item inventory of humor uses in everyday life adapted from Graham et al. (1992). This inventory groups humor uses into affiliative and non-affiliative functions, and for each type, it includes items that pertain to humor use at the individual level, the interpersonal level, and the societal level. Vaid et al. (2004) found that all groups reported higher affiliative uses of humor (e.g., to be playful, affectionate, reduce conflict) than non-affiliative uses (e.g., to manage one’s anxieties, to express hostility, to expose injustices). Differences in perception of one’s own humor use vs. humor use by one’s culture were minimal in the African-American and Euro-American groups, but were significant in the two immigrant groups. The Asian American sample showed the largest difference between self and culture ratings of all four groups, suggesting that their perception of how their culture of origin uses humor was very different from how they themselves used humor. 

Looking more closely at Asian vs. Euro-American cultural differences in humor judgments, Vaid et al. (2008) compared monolingual English speakers from the US, monolingual Korean speakers in South Korea, and Korean-English bilinguals in the US. Participants were to rate narratives presented in English or Korean depicting embarrassing everyday predicaments (such as accidentally spilling soup on someone at a restaurant, or finding that someone else is wearing the same outfit as oneself). The Euro-American sample judged the scenarios as more amusing than did the two Korean groups, who in turn found them to be more embarrassing. Bilinguals judged predicaments as more amusing when framed with English emotion labels. In a follow-up study asking participants how they would respond if they were in the depicted situation, Euro-Americans were more likely to use humor to defuse embarrassing situation whereas those from Korea were more likely to apologize or to act as if nothing happened. This study illustrates that cultural norms shape perceptions of the humorousness of a situation and can make humor more or less likely as a response to embarrassment. More recently, a study comparing a Canadian and a Hong Kong sample found that the groups differed in their evaluation of humor: humor was perceived as a positive and common disposition by the Canadians, but as a particular disposition associated with humorists by the Chinese sample (Yue et al., 2016). 

From this brief discussion, it should be apparent that humor in interpersonal communication has a variety of uses and that these uses in turn may vary as a function of cultural schemas about when humor is appropriate or not, or even what types of humor are permissible. Humor researchers have sought to develop measures that capture the many different facets of the construct of humor in order to facilitate group comparisons. This is particularly challenging given that humor is a complex and highly contextual construct, making it important that multiple and converging measures are used across a variety of groups and settings. 

A self-report measure of humor was developed in 2003 by Martin et al. to study individual differences in styles of humor use in daily life and their relationship to psychological well-being (Martin et al., 2003). This instrument, termed the humor styles questionnaire (HSQ) (see also Martin, 2015), posits four dimensions of humor that may differ across individuals or groups: affiliative, self-enhancing, aggressive, and self-defeating. *Affiliative humor*, as conceptualized by Martin et al. (2003, p. 53), is “an essentially non-hostile, tolerant use of humor that is affirming of self and others and presumably enhances interpersonal cohesiveness and attraction”. Individuals who use this humor style are likely to say funny things, engage in witty banter, and put others at ease. *Self-enhancing humor* refers to a style of humor in which a humorous perspective is held as a coping mechanism in the face of adversity; its focus is to regulate negative emotion through humorous perspective-taking. *Aggressive humor* involves the use of sarcasm, ridicule, or disparaging humor. *Self-defeating humor* involves “attempts to amuse others by doing or saying funny things at one’s own expense as a means of ingratiating oneself or gaining approval”, (Martin et al., 2003, p. 54) or using humor to hide one’s underlying feelings.

The HSQ is unique because it identifies humor styles that theoretically impact interpersonal wellbeing in adaptive (self-enhancing and affiliative) and maladaptive (aggressive and self-defeating) ways, however whether these domains have negative and positive impacts on well-being has been a subject of debate (Heintz & Ruch, 2015, 2016). For example, the self-defeating humor, which is proposed to negatively impact well-being, may refer to the ability to laugh at yourself which can be adaptive and beneficial (Ruch & Heintz, 2017). Additionally, scale items on the HSQ contain humor-related and non-humor related content (Ruch & Heintz, 2017). For example, the self-defeating humor item “I let people laugh at me or make fun at my expense more than I should” contains non-humor content (“more than I should”).

The HSQ was developed through a pool of items that were validated using mostly Canadian undergraduate psychology students. However, the HSQ has been examined globally across many different countries. Suh (2006) cross-validated the HSQ, translated into Korean, in a Korean sample and found similar scale reliabilities and factor loadings. In this study, Korean participants scored lower than US participants on all four humor scales of the HSQ. Schermer et al. (2023) collected responses to the HSQ in 21 different languages across 28 different countries. They found that, across all the countries, affiliative humor typically had the highest scores, and that self-enhancing humor was significantly higher in the American and Canadian samples, particularly among men. Yee and Lee (2024) found that, in a sample from Malaysia, schadenfreude (gaining pleasure from others misery) was significantly associated with self-defeating and aggressive humor.

From this overview we see that humor styles and conversation styles vary across different cultural groups, reflecting social norms about what is appropriate or inappropriate to say. What few studies have addressed is the potential ways in which conversational style, and in particular, a preference for indirectness in the expression or interpretation of linguistic meaning, may find resonance with particular forms of humor in conversation. Conversational indirectness and certain forms of humor involve a tension between wanting to convey something that may be seen as face-threatening and looking for a way to convey it that is both palatable and creative.

## 4. The Present Study

The present study sought to jointly examine humor style and conversational indirectness. We examined these constructs in a sample of Korean-English bicultural bilinguals whose first language (L1) is Korean, and a sample of Americans whose L1 is English. We were interested in exploring three issues: (1) how conversational indirectness in interpretation and production may vary across these two groups, (2) how self-reported preference for the four humor styles of the HSQ may vary across the groups, and (3) whether indirectness in conversational style is correlated with a preference for a particular style of humor. 

Based on previous research we expected that Korean participants would report higher levels of indirectness in both interpretation and production compared to American participants, but that they would show lower levels of all four styles of humor relative to American participants. Our third question was exploratory and we did not have a specific prediction about how conversational indirectness may be associated with particular styles of humor. 

## 5. Method

### 5.1. Participants 

Data were collected from a total of 98 participants recruited from Texas A&M University as part of a larger project on cultural differences in humor use (see Vaid et al., 2008). After removing those who did not complete all questionnaire items and those whose first language was not either English or Korean, we were left with a total of 88 participants, 56 English L1 speakers (41 female, 15 male) and 32 Korean first language speakers (17 female, 15 male). The English L1 group’s ages ranged from 20 to 30 years (*M* = 21.71, *SD* = 1.45) with one participant’s age not listed, while the Korean group’s ages ranged from 18 to 36 years (*M* = 29.84, *SD* = 3.62) with one participant’s age not listed.

All Korean L1 participants were Korean-English bilinguals who had spent an average of 4.4 years in the US and almost all considered Korean to be their most effective language for communication. On a self-report measure of cultural identity, the majority of the Korean L1 participants reported their cultural identity as more Korean than American and reported keeping their Korean and American cultural spheres separate.

### 5.2. Materials and Procedure

To assess conversational indirectness tendency, we used Holtgraves’s (1997) conversational indirectness scale (CIS). The CIS measures conversational indirectness on two domains, production (a person’s tendency to convey meaning indirectly) and interpretation (a person’s tendency to look for indirect meaning in other’s remarks). The CIS has 19 items, 10 for the interpretation subscale and 9 for the production subscale. Sample items from each subscale can be viewed in Table 1. On each item, participants were to indicate their extent of agreement with the statement, on a scale of 1 (completely disagree) to 7 (completely agree). To allow for direct comparisons across the two subtypes, mean responses were calculated per subscale per group. Higher mean scores on this scale would indicate a greater preference for an indirect conversational style in expression and/or interpretation of meaning. 

To assess participants’ preferred humor styles, we used the humor styles questionnaire (HSQ) by Martin et al. (2003). As noted earlier, this measure conceptualizes humor style into four distinct types: self-enhancing (humor that enhances the self by having a generally humorous outlook on life), affiliative (using humor to enhance and facilitate social relationships in a self-accepting manner), aggressive (humor that enhances the self at the expense of or without regard to others), and self-defeating (using humor to enhance social relationships at the expense of the self). Sample items for each dimension can be viewed in Table 1. The HSQ has 32 items total, with 8 items for each dimension. Like the CIS, the HSQ is a self-report questionnaire, and scores were calculated as mean scores for each subscale. Higher scores indicate higher preference for each humor dimension for an individual. 

For both the CIS and HSQ, Korean participants were asked to rate each statement on a 1 to 7 Likert scale (1 = disagree, 7 = agree) as it applied to their conversational behavior when speaking with other members of their language community. American participants were asked to rate each statement on a similar 1 to 7 Likert scale. All participants first completed the CIS and then the HSQ. Participants also completed a demographic questionnaire. All measures were completed in English on printed questionnaires. 

### 5.3. Statistical Analysis

All statistical analyses were conducted using R Statistical Software (v4.4.2; R Core Team, 2024). Descriptive statistics, including means and standard deviations, were calculated for the conversational indirectness scale (CIS) and humor styles questionnaire (HSQ) subscales for each language group. These descriptive summaries were obtained using the summary function in R.

To examine group differences in conversational indirectness, a 2 (Language Group: English vs. Korean) × 2 (Communication Mode: Production vs. Interpretation) repeated-measures ANOVA was conducted. The ANOVA model was computed using the aov function, with error terms specified to account for repeated measures.

To compare humor styles across language groups, four independent *t*-tests were performed to assess differences in affiliative, self-enhancing, aggressive, and self-defeating humor between English and Korean speakers. The *t*-test function was used for these comparisons, and a Bonferroni correction was applied using the p.adjust function to account for multiple comparisons and control the familywise error rate.

To explore the relationships between conversational indirectness and humor styles within each language group, Pearson correlation matrices were computed separately for English and Korean speakers using the cor function. Finally, to determine whether the strength of these correlations significantly differed between language groups, Zou’s (2007) confidence interval method was applied. These cross-group correlation comparisons were performed using the cocor.indep.groups function from the cocor R package (v1.1.4; Diedenhofen & Musch, 2015), allowing for an assessment of whether the relationships between conversational indirectness and humor styles varied systematically across cultures. All statistical tests were conducted using an alpha level of α = 0.05 to determine significance.

## 6. Results

Three sets of analyses were conducted, one focusing on conversational indirectness as a function of cultural group, the second on humor style as a function of cultural group, and the third on correlations between conversational style and humor style in each group. 

### 6.1. Conversation Style

**Figure 1:**
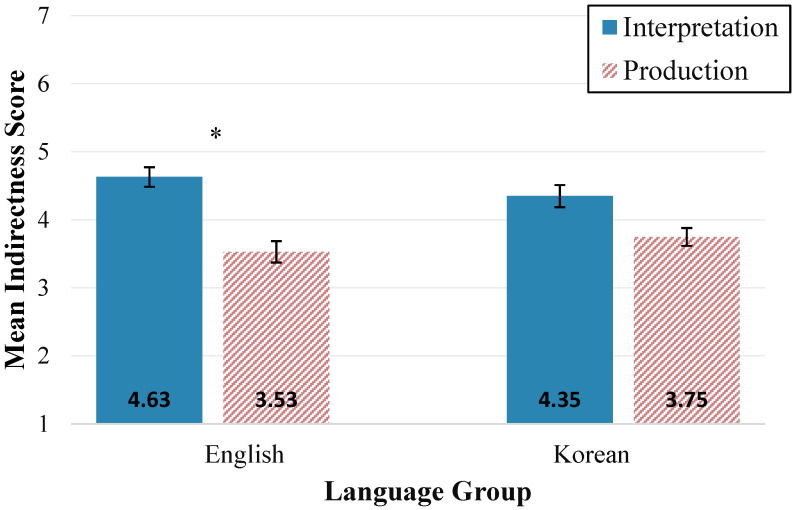
Mean conversational indirectness scores (CISs) by first language Group. Note. Variability is expressed as standard error. Communication mode comparisons that are significant at *p* < 0.001 are indicated with an asterisk.

In order to understand potential language group differences in preference for conversational indirectness, a 2(Language Group: English × Korean) by 2(Communication Mode: production × interpretation) analysis of variance was conducted, with repeated measures on communication mode. There was no main effect for language group (*F*(1, 86) = 0.25, *p* = 0.620). There was a significant main effect of communication mode on indirectness scores (*F*(1, 86) = 38.64, *p* < 0.001), where indirectness scores on interpretation (*M* = 4.45, *SD* = 1.04) were significantly greater than scores on production (*M* = 3.50, *SD* = 1.05). There was also a significant interaction between language group and communication mode (*F*(1, 86) = 5.45, *p* = 0.022). Post hoc comparisons using the Tukey HSD *t*-test with Bonferroni correction indicated that the mean indirectness score for the English interpretation (*M* = 4.63, *SD* = 1.08) was significantly higher than the English production (*M* = 3.53, *SD* = 1.18), *t*(86) = 7.09, *p* < 0.001, but there was no significant difference in Korean interpretation vs. Korean production mean scores (see Figure 1).

### 6.2. Humor Style

**Figure 2:**
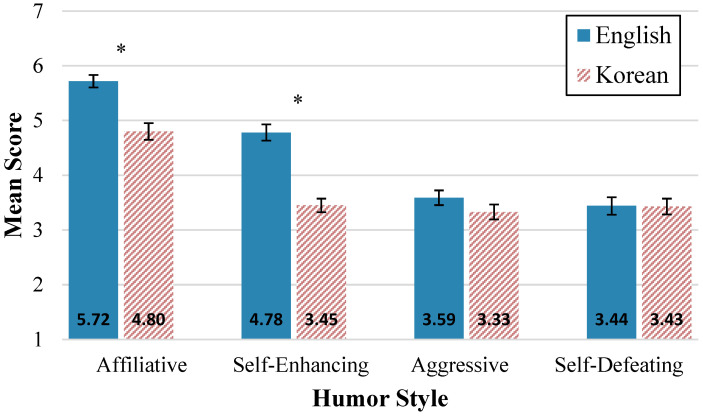
Mean responses on humor styles questionnaire by first language. Note. Variability is expressed by standard error bars. Group comparisons that are significant at *p* < 0.01 are indicated with an asterisk.

In order to assess language group differences on the four HSQ domains, we conducted four independent *t*-tests, adjusting the alpha level using the Bonferroni correction to account for multiple comparisons. All variables passed Levene’s test for homogeneity of variance with the exception of self-defeating humor (*F*(1, 86) = 5.25, *p* = 0.024). Results indicated that the average score for affiliative humor was significantly higher in the English L1 group (*M* = 5.72, *SD* = 0.86) than Korean L1 group (*M* = 4.80, *SD* = 0.87), *t*(86) = 4.81, *p* = 0.004. The self-enhancing humor score was also significantly higher in the English L1 group (*M* = 4.78, *SD* = 1.11) compared to the Korean L1 group (*M* = 3.45, *SD* = 0.71), *t*(86) = 6.14, *p* = 0.004. However, we did not find significant differences between our language groups for aggressive humor (*t*(86) = 1.26, *p* = 0.848) and self-defeating humor (*t*(86) = 0.03, *p* = 0.999). See Figure 2 for mean HSQ scores for each language group.

### 6.3. Conversation Style in Relation to Humor Style

Pearson correlations were computed separately for English and Korean speakers to examine the relationships between conversational indirectness and humor style. The correlation matrices for each group are presented in Table 2 (English first-language speakers) and Table 3 (Korean first-language speakers), providing an overview of how conversational indirectness and humor styles are related within each language group.

### 6.4. English Language Group 

A summary of the correlation matrix for this group is provided in Table 2. A significant positive correlation was found between indirectness in interpretation and indirectness in production. This is consistent with previous findings by Holtgraves (1997) and others. A significant positive correlation was found between affiliative humor and self-enhancing humor, and between self-defeating humor and self-enhancing humor. This, too, has previously been reported in the literature, particularly for the Canadian and American samples most often studied (Martin et al., 2003). 

Three correlations were found between conversation indirectness and humor style. An aggressive humor style showed a significant positive correlation with indirectness in production. A self-defeating humor style also showed a significant positive correlation with indirectness in production. In addition, a self-defeating humor style showed a significant positive correlation with indirectness in interpretation and production. Lastly, self-defeating and self-enhancing humor showed a significant positive correlation.

### 6.5. Korean Language Group 

Unlike the English L1 group, the Korean L1 sample showed no significant correlation between conversational indirectness in interpretation and production. Nor did they show a correlation between affiliative and self-enhancing humor. Instead, they showed a significant positive correlation between aggressive humor and self-defeating humor. 

With respect to correlations between conversational indirectness and humor style, this group showed a significant positive correlation between aggressive humor and indirectness in production.

### 6.6. Language Group Comparisons 

The correlations were formally compared across language groups using Zou’s (2007) confidence interval approach. Results revealed two significant differences in correlation strengths between groups.

First, conversational indirectness in interpretation was significantly related to self-enhancing humor, but in opposite directions for English and Korean speakers. English speakers showed a positive correlation between these two variables (*r* = 0.24), whereas Korean speakers showed a negative correlation (*r* = −0.27). The difference between these correlations was statistically significant (Δ*r* = 0.51, 95% CI = [0.06, 0.88]). This suggests that indirect communication may promote self-enhancing humor for English speakers but suppress it for Korean speakers.

Second, a significant difference was found for conversational indirectness in interpretation and aggressive humor. English speakers exhibited a weak positive correlation (*r* = 0.10), while Korean speakers showed a moderate negative correlation (*r* = −0.36). The difference between these correlations was also statistically significant (Δ*r* = 0.45, 95% CI = [0.02, 0.83]), indicating that more indirect communication in interpretation is associated with lower aggressive humor among Korean speakers but not among English speakers.

Other correlation comparisons did not reach statistical significance (*p* > 0.05), meaning that while numerical differences were observed across groups, they may not reflect meaningful population differences. These include the relationship between conversational indirectness in production and aggressive humor (Δ*r* = −0.15, 95% CI = [−0.51, 0.24]), as well as between conversational indirectness and self-defeating humor (Δ*r* = 0.05, 95% CI = [−0.34, 0.47]). Additionally, the relationships between different humor styles (e.g., affiliative and self-enhancing humor) were similar across groups, suggesting that humor style preferences may function similarly regardless of cultural background.

## 7. Discussion

Our study had three objectives: to investigate cultural group differences in conversational indirectness, cultural group differences in humor style, and the relationship between conversational indirectness and humor style within and across groups.

### 7.1. Conversational Indirectness

With respect to the first objective, we hypothesized that conversational indirectness would be higher in the Korean group compared to the American group. However, we did not find support for an overall group difference. Instead, we found a group by mode interaction, which showed that higher scores for indirectness characterized the interpretation mode relative to the production mode, but this effect was restricted to the English L1 group. Korean L1 participants did not show a significant difference between communication modes.

The lack of group differences in conversational indirectness goes against a previous finding by Holtgraves (1997) in which Korean participants tested in South Korea scored higher on conversational indirectness compared to American participants. The discrepancy between our results and those of Holtgraves may in part be attributed to the fact that our Korean participants were recruited from an international student population in the US, and were tested in English, their second language. The fact that our Korean participants had been living in the US for a few years, even though they self-reported their cultural identity to be more Korean than American, may have impacted their conversational style in English to be more like the L1 English speakers in this study. These factors may have contributed to increase response similarities between our two language groups. This interpretation is supported by two studies. Ross et al. (2002) found that Chinese-English bilinguals who were randomly assigned to provide self-perceptions in Chinese or in English showed more collective self-statements and greater agreement with Chinese cultural values when responding in Chinese. In a separate study, Marian and Kaushanskaya (2004) found that Russian-English bilinguals’ autobiographical memories were influenced by cultural schemas: when speaking a language associated with a more collectivist culture, the narratives produced were more collectivist, regardless of the language of encoding. These studies highlight the importance of language as a vehicle for culture. 

Given that we did not compare performance of our Korean sample in Korean vs. English, we cannot be sure if the lack of group differences we observed in conversational indirectness reflects the English-dominant milieu in which they were living. At the very least, our results suggest that under certain conditions even members of a culture considered more collectivist can show comparable levels of directness to that of members of a culture considered more individualist. 

What is interesting, nonetheless, is that the Korean sample showed no difference in indirectness tendencies in interpretation vs. production, whereas the American sample showed a preference for indirectness in interpretation. Thus, the American sample was more likely to look for hidden meanings in the remarks of others but less likely to produce indirect remarks themselves. Furthermore, the groups differed in one other respect: a significant correlation was found in the American group between indirectness in interpretation and indirectness in production. This positive correlation was an anticipated result, and corroborates Holtgraves’s (1997) finding that people who interpret more indirect meanings also tend to produce more indirect remarks. By contrast, the Korean group did not show a correlation between these two modes although the correlational analyses (discussed below) suggest that this group did respond differently on these two aspects of conversational indirectness even if they did not show a correlation between them. 

### 7.2. Humor Style

With respect to our second objective, we hypothesized that the American sample would show higher levels of humor use for all four tested dimensions, the two so-called positive dimensions of affiliative and self-enhancing humor styles, and the two so-called negative dimensions of self-defeating and aggressive humor. Our results bore this out only for the two positive dimensions. Korean participants had significantly lower scores on affiliative and self-enhancing humor compared to English L1 participants. 

In a previous study, Suh (2006) had found that Korean participants who had been administered the humor style questionnaire in Korean scored lower than American participants on all four humor dimensions. Again, given that our Korean participants completed the humor questionnaire in English may have contributed to the pattern of results. Still, it is noteworthy that our Korean sample did show a lower level of use of humor for affiliative and self-enhancing purposes, compared to the American sample. These findings support other studies that suggest both that these positive dimensions are more highly endorsed by members of a wide range of languages studied, and that American participants tend to show higher levels of humor use relative to other groups on the positive dimensions (Schermer & Kfrerer, 2020). 

As expected, we also saw correlations between different styles of humor. The HSQ’s “positive” humor styles (affiliative and self-enhancing) were correlated positively with each other in our English L1 group analyses. The HSQ’s “negative” humor styles (aggressive and self-defeating) were correlated in our Korean L1 group analyses. These results were also expected, as both sets of humor dimensions have conceptual similarities (Martin et al., 2003). Somewhat unexpectedly, self-enhancing and self-defeating humor, which share less conceptual similarities, were correlated positively in our English language group. Both these styles of humor focus on “self” uses of humor in theoretically opposing ways (enhancing the self vs. putting down the self for relational connection), however this positive correlation indicates that having a generally humorous outlook on life and using humor at the expense of the self are not opposing but related to each other in our English language group. 

These language group differences are reflected in a study by Dunman and Wang (2024), who found that, in their sample, Americans showed a stronger positive bias in self-related event memory recall compared to East Asians. Our English L1 participants had higher scores on both positive humor domains and these domains were positively correlated in this group. Korean L1 participants had both negative humor domains positively correlated with each other. Together, these findings may provide insight into differences in humor preferences and/or positive biases between these two groups and could possibly be extrapolated to encompass a difference between the two cultures. Perhaps American culture and/or the English language has a stronger positive bias than Korean culture/language. 

However, there are other confounding factors that these group differences may be attributed to. Korean participants completed the questionnaires in their non-native language. Second language learners often have more difficulty expressing humor through their non-native language (Vaid, 2006), and this may be reflected more in relational “positive” uses of humor than in “negative” uses of humor. Future studies should take this into account and compare bilinguals in both their native and non-native languages to gain a fuller understanding of where group differences are derived from. 

Additionally, the Korean L1 participants were overall older than the English L1 participants, so these group differences may be attributed in some part to participants’ age. Support for age differences comes from Martin et al.’s (2003) study, where American participants age 14 to 19 scored higher on affiliative humor than participants age 25 to 87; for further discussion of the effects of aging on humor processing, see (Bischetti et al., 2023), and (Greengross, 2013). Further research is needed to investigate these humor style differences and how age plays a role in humor preferences across language/cultural groups. 

Across both language groups, affiliative humor was the most popular humor style with the highest scores. This suggests that, despite group differences, uses of humor to relate to others were the most highly reported. This finding is consistent with Schermer et al. (2023), who reported that affiliative humor was the most preferred humor style across 28 different countries. Moreover, according to Ruch and Heintz (2017), affiliative humor has the highest construct validity of all the humor domains in the HSQ, meaning that it contains the most humor-relevant content compared to the other humor domains which contain more non-humor-relevant content. This may be why it was the most popularly reported form of humor style in many different countries (Schermer et al., 2023) and in our sample.

### 7.3. Conversational Indirectness in Relation to Humor Style

Finally, our study explored associations between conversational indirectness and humor style. For this aspect of our study, we did not make specific predictions. Nevertheless, we found an interesting pattern of somewhat overlapping associations. What is noteworthy is that affiliative humor did not show any correlation with indirectness in interpretation or production in either group. This suggests that it may be safer to engage in this type of humor in social settings when there is no other message being conveyed besides social connection through humor. Interestingly, when significant correlations were obtained, they were with the two “negative” dimensions of humor, namely, self-defeating and aggressive humor. Specifically, both cultural groups showed a significant positive correlation between conversational indirectness in production and scores on aggressive humor. Given that aggressive humor refers to humor that teases or insults others, it makes sense that those who reported using such humor also reported higher levels of indirectness in production. The correlation suggests that when the intended meaning of a message is a criticism or put-down of some sort, a preferred communicative strategy is to be indirect in expressing it; communicating it through humor may be a safer way of expressing negative meaning. Of course, it is not always the case that expressing criticism through humor is a safe strategy as it can sometimes backfire.

Additionally, in a study by Ruch and Heintz (2017), the construct relevance of the aggressive humor scale was only partially supported, indicating that this scale contained humor-relevant and non-humor relevant elements. Indirect production in our study may be related to either or both the humor-relevant content of the aggressive humor scale or the non-humor related content. For aggressive humor, if indirect production associated with humorous content, playfully teasing or making fun of others may be related to more indirect forms of communication. If it is related to non-humorous content, it may be that being more indirect is associated with the aggressive scale’s humor-irrelevant content.

Similarly, the construct relevance for the self-defeating humor scale was not supported in Ruch and Heintz’s (2017) study, indicating that this scale aligns most with its non-humorous elements. So, for self-defeating humor in our English group, indirect production and interpretation are likely associated with elements that are less related to humor. Future studies on conversational indirectness and humor style should use Ruch and Heintz’s (2017) humor-only version of the HSQ to see if these findings are replicable in a humor-only version of the HSQ. For further discussion regarding the use of the HSQ for individual differences in style profiles, please see Galloway (2023).

Interestingly, a negative correlation was observed in the Korean sample between indirectness in interpretation and aggressive humor. While not significant, this pattern of results for the Korean sample suggests that, at least with respect to aggressive humor, they showed a difference in their responses to indirectness in interpretation and indirectness in production.

Relatedly, a significant correlation was observed in the English L1 group between self-defeating humor and conversational indirectness in production and interpretation. This correlation suggests that individuals who are more likely to express meanings indirectly are also more likely to engage in humor that is self-deprecating, as a way to ingratiate themselves with others and gain their approval. 

Finally, our study uncovered group differences in the relationship between self-enhancing humor and conversation indirectness in interpretation, with the English group showing a positive correlation and the Korean group a negative one. Moreover, conversational indirectness in interpretation showed a stronger association with aggressive humor in the English group than in the Korean group. 

Taken together, the observed associations suggest that aggressive and self-defeating humor styles, or humor that makes fun of others or makes fun of oneself, may have “indirect” elements that resonate with an indirect mode of communicating than humor that seeks simply to bring people closer. These “negative” humor styles of self-defeating or aggressive humor may be used by some people to convey covert meanings below the surface of the humorous remark or action. However, some aggressive humor may have other intentions that are not directly stated. For example, someone who teases others may do so with positive intentions. Our study also suggests that the relationship between humor style and conversation style is not uniform across cultural groups.

### 7.4. Limitations

One limitation of the study was that our English L1 participants were not asked to imagine a specific context for the questions asked on either questionnaire. The context for our Korean L1 participants was “when speaking with other members of your language community”, but this was not specified further. Participants across both language groups may have envisioned different contexts of conversation (work/school/home, parents/friends/etc.). This may have an impact on reported scores as seen in Sanchez-Burks et al. (2003); see Gheorghe and Curseu (2024) for a review of the effects of social context on humor in groups). 

Also, as noted earlier, the fact that the Korean participants were in an English-dominant environment at the time of testing may have contributed in some part to the similarities observed with the American sample. Additionally, the variability across groups in age range may have contributed to some of the group differences noted. Despite these limitations, our study points to an intriguing pattern of associations between humor style and conversational indirectness with some points of convergence and divergence across groups differing in their first language. 

## 8. Conclusions

Our findings offer preliminary evidence for a relationship between indirectness as a communication mode and a preference for certain types of humor. Across both language groups, a higher tendency to produce remarks with indirect meanings was associated with a higher tendency to use either self-defeating or aggressive humor. The association between the production of indirect remarks and aggressive and self-defeating humor provides a preliminary jumping off point to further investigate the association between humor and indirectness. It will be important in future research to develop other ways to probe the nature of the relationship between indirectness in expression of a meaning and the use of so-called negative uses of humor in conversation. 

## Figures and Tables

**Table 1:** Sample Items from Each Measure.

Domain Subscale	Sample Item	Sample Item (Reverse-Worded)
Conversational Indirectness Scale		
Production	My remarks often have more than one meaning.	Most of what I say can be taken at face value, and there is no need to look for a deeper meaning.
Interpretation	In order to understand someone’s remark, I will often look at why it was said rather than what was said.	I don’t usually look for deeper meanings in the remarks of others.
Humor Styles Questionnaire		
Self-enhancing	Even when I am by myself, I’m often amused by the absurdities of life.	If I am feeling sad or upset, I usually lose my sense of humor.
Affiliative	I laugh and joke a lot with my closest friends.	I don’t often joke around with my friends.
Aggressive	If someone makes a mistake, I will often tease them about it.	I never participant in laughing at others even if all my friends are doing it.
Self-defeating	I will often get carried away in putting myself down if it makes my family or friends laugh.	I don’t often say funny things to put myself down.

Note. Sample items from the CIS (Holtgraves, 1997) and HSQ (Martin et al., 2003).

**Table 2:** Correlation matrix for English first-language group.

Variables	CIS Interpretation	CIS Production	HSQ Affiliative	HSQ Self-Enhancing	HSQ Aggressive
CIS Production	0.44 **	__			
HSQ Affiliative	0.04	0.12	__		
HSQ Self-Enhancing	0.24	0.19	0.45 **	__	
HSQ Aggressive	0.10	0.31 *	0.19	0.19	__
HSQ Self-Defeating	0.31 *	0.35 *	0.24	0.30 *	0.18

Note. *N* = 56. * *p* < 0.05. ** *p* < 0.01.

**Table 3:** Correlation matrix for Korean first-language group.

Variables	CIS Interpretation	CIS Production	HSQ Affiliative	HSQ Self-Enhancing	HSQ Aggressive
CIS Production	0.12	__			
HSQ Affiliative	0.05	−0.14	__		
HSQ Self-Enhancing	−0.27	0.22	0.23	__	
HSQ Aggressive	−0.36	0.46 *	0.09	0.19	__
HSQ Self-Defeating	0.26	0.36	0.30	0.19	0.53 **

Note. *N* = 32. * *p* < 0.05. ** *p* < 0.01.

## Data Availability

Data is contained within the article.

## References

[B1-behavsci-15-00252] Bischetti L., Ceccato I., Lecce S., Cavallini E., Bambini V. (2023). Pragmatics and theory of mind in older adults’ humor comprehension. Current Psychology.

[B2-behavsci-15-00252] Brown P., Levinson S. C. (1987). Politeness: Some universals in language usage.

[B3-behavsci-15-00252] Chen X., Wang J. (2021). First order and second order indirectness in Korean and Chinese. Journal of Pragmatics.

[B4-behavsci-15-00252] Crawford M., Gressley D. (1991). Creativity, caring, and context: Women’s and men’s accounts of humor preferences and practices. Psychology of Women Quarterly.

[B5-behavsci-15-00252] Cunningham D., Vaid J., Chen H. C., Cook V., Bassetti B. (2010). Yo no lo tire, se cayo solito, ‘I did not throw it, it just fell down’: Interpreting and recounting accidental events in Spanish and English. Language and bilingual cognition.

[B6-behavsci-15-00252] Diedenhofen B., Musch J. (2015). Cocor: A comprehensive solution for the statistical comparison of correlations. PLoS ONE.

[B7-behavsci-15-00252] Dunman C., Wang Q. (2024). Culture and memories of self-conscious emotions. Annual Meeting of the Psychonomic Society.

[B8-behavsci-15-00252] Dynel M. (2009). Beyond a joke: Types of conversational humour. Language and Linguistics Compass.

[B9-behavsci-15-00252] Galloway G. (2023). The Humor Styles Questionnaire: A critique of scale construct validity and recommendations regarding individual differences in style profiles. Humor.

[B10-behavsci-15-00252] Gheorghe A., Curseu P. L. (2024). From I to we in humor research: A systematic review of the antecedents and consequences of humor in groups. Humor.

[B11-behavsci-15-00252] Goffman E. (1967). Interaction ritual: Essays on face-to-face interaction.

[B12-behavsci-15-00252] Graham E., Papa M., Brooks G. (1992). Functions of humor in conversation: Conceptualization and measurement. Western Journal of Communication.

[B13-behavsci-15-00252] Greengross G. (2013). Humor and aging: A mini-review. Gerontology.

[B14-behavsci-15-00252] Hall E. T. (1976). Beyond culture.

[B15-behavsci-15-00252] Hara K., Kim M.-S. (2004). The effect of self-construals on conversational indirectness. International Journal of Intercultural Relations.

[B16-behavsci-15-00252] Heintz S., Ruch W. (2015). An examination of the convergence between the conceptualization of humor styles and the measurement of humor styles: A study of the construct validity of the Humor Styles Questionnaire. Humor.

[B17-behavsci-15-00252] Heintz S., Ruch W. (2016). Reply to Martin (2015): Why our conclusions hold. Humor.

[B18-behavsci-15-00252] Holtgraves T. (1997). Styles of language use: Individual and cultural variability in conversational indirectness. Journal of Personality and Social Psychology.

[B19-behavsci-15-00252] Jiang T., Li H., Ho Y. (2019). Cultural differences in humor perception, usage, and implications. Frontiers in Psychology.

[B20-behavsci-15-00252] Lopez B. G., Vaid J., Attardo S. (2017). Psycholinguistic approaches to humor. The routledge handbook of language and humor.

[B21-behavsci-15-00252] Lu J. G. (2023). Cultural differences in humor: A systematic review. Current Opinion in Psychology.

[B22-behavsci-15-00252] Marian V., Kaushanskaya M. (2004). Self-construal and emotion in bicultural bilinguals. Journal of Memory and Language.

[B23-behavsci-15-00252] Marti L. (2006). Indirectness and politeness in Turkish-German bilingual and Turkish monolingual requests. Journal of Pragmatics.

[B24-behavsci-15-00252] Martin R. A. (2015). On the challenges of measuring humor styles: Response to Heintz and Ruch. Humor.

[B25-behavsci-15-00252] Martin R. A., Puhlik-Doris P., Larsen G., Gray J., Weir K. (2003). Individual differences in uses of humor and their relation to psychological well-being: Development of the Humor Styles Questionnaire. Journal of Research in Personality.

[B26-behavsci-15-00252] Merken R., Taras V., Steel P. (2014). State of the art themes in cross-cultural communication research: A systematic and meta-analytic review. International Journal of Intercultural Relations.

[B27-behavsci-15-00252] Miyahara A. (1996). Philosophical issues in communication research: Cross-cultural perspectives on competence.

[B28-behavsci-15-00252] Norrick N. (2003). Issues in conversational joking. Journal of Pragmatics.

[B29-behavsci-15-00252] R Core Team (2024). R: A language and environment for statistical computing.

[B30-behavsci-15-00252] Ross M., Xun W. Q., Wilson A. (2002). Language and the bicultural self. Personality and Social Psychology Bulletin.

[B31-behavsci-15-00252] Ruch W., Heintz S. (2017). Experimentally manipulating items informs on the (limited) construct and criterion validity of the Humor Styles Questionnaire. Frontiers in Psychology.

[B32-behavsci-15-00252] Sanchez-Burks J., Lee F., Choi I., Nisbett R., Zhao S., Koo J. (2003). Conversing across cultures: East-West communication styles in work and nonwork contexts. Journal of Personality and Social Psychology.

[B33-behavsci-15-00252] Schermer J. A., Kfrerer M. (2020). Humor style differences across four English-speaking countries. Humor.

[B34-behavsci-15-00252] Schermer J. A., Rogoza R., Kwiatkowska M. M., Kowalski C. M., Aquino S., Ardi R., Bolló H., Brankovic M., Chegeni R., Crusius J., Doroszuk M., Enea V., Truong T. K. H., Ilisko D., Jukic T., Kozarevic E., Kruger G., Kurtic A., Lange J., Krammer G. (2023). Humor styles across 28 countries. Current Psychology.

[B35-behavsci-15-00252] Suh S. (2006). Relationship between humor styles and health behaviors in Korea and the United States: A cross-cultural comparison. Master’s thesis.

[B36-behavsci-15-00252] Tannen D. (1981). Indirectness in discourse: Ethnicity as conversation style. Discourse Processing.

[B37-behavsci-15-00252] Tannen D. (1984). Conversational style: Analyzing talk among friends.

[B38-behavsci-15-00252] Ting-Toomey S., Kim Y., Gudykunst W. (1988). Intercultural conflict style. Theories in intercultural communication.

[B39-behavsci-15-00252] Tosun S., Faghihi N., Vaid J. (2018). Is an ideal sense of humor gendered? A cross-national study. Frontiers in Psychology.

[B40-behavsci-15-00252] Triandis H. C. (1989). The self and social behavior in differing cultural contexts. Psychological Review.

[B41-behavsci-15-00252] Vaid J., Pavlenko A. (2006). Joking across languages: Perspectives on humor, emotion, and bilingualism. Bilingual minds: Emotional experience expression, and representation.

[B42-behavsci-15-00252] Vaid J., Choi H., Chen H., Friedman M. (2008). Perceiving and responding to embarrassing predicaments across languages: Cultural influences on the emotion lexicon. The Mental Lexicon.

[B43-behavsci-15-00252] Vaid J., Choi H., Martinez F., Chen H. C. (2004). Perception of humor uses for self vs. culture across four groups. Annual Meeting of the Association for Psychological Science.

[B44-behavsci-15-00252] Vaid J., Ramachandran V. S., Blakemore C., Jennett S. (2001). Laughter and humour. The Oxford Companion to the Body.

[B45-behavsci-15-00252] Wang Q. (2016). Why should we all be cultural psychologists? Lessons from the study of social cognition. Perspectives on Psychological Science.

[B46-behavsci-15-00252] Wang Q., Ross M. (2005). What we remember and what we tell: The effects of culture and self-priming on memory representations and narratives. Memory.

[B47-behavsci-15-00252] Yee J. W., Lee S. L. (2024). The Dark Trait traits, humor styles, and *schadenfreude*: Others’ misery as the devil’s laughing stocks. The Japanese Psychological Association.

[B48-behavsci-15-00252] Yu K.-A. (2011). Culture-specific concepts of politeness: Indirectness and politeness in English, Hebrew, and Korean requests. Intercultural Pragmatics.

[B49-behavsci-15-00252] Yue X., Jiang F., Lu S., Hiranandani N. (2016). To be or not to be humorous? Cross cultural perspectives on humor. Frontiers in Psychology.

[B50-behavsci-15-00252] Zou G. Y. (2007). Toward using confidence intervals to compare correlations. Psychological Methods.

